# Liquid Biopsy for Minimal Residual Disease Assessment in Endometrial and Cervical Cancers: Molecular Rationale, Clinical Evidence, and Translational Barriers

**DOI:** 10.3390/ijms27167155

**Published:** 2026-08-10

**Authors:** Ludovica Pepe, Valeria Zuccalà, Walter Giuseppe Giordano, Giordana Di Mauro, Vincenzo Cianci, Cristina Mondello, Massimiliano Berretta, Vincenzo Fiorentino, Antonio Ieni

**Affiliations:** 1 Anatomic Pathology Unit, Department of Human Pathology in Adult and Developmental Age “Gaetano Barresi”, University of Messina, 98125 Messina, Italy; ludopepe97@gmail.com (L.P.); valeria.zuccala@unime.it (V.Z.); 2 PhD Program in Translational Molecular Medicine and Surgery, Department of Biomedical, Dental, Morphological and Functional Imaging Sciences, University of Messina, 98125 Messina, Italy; waltergiordano1997g@gmail.com; 3 Division of Medical Oncology, Department of Clinical and Experimental Medicine, University of Messina, 98125 Messina, Italy; giordana.dimauro@studenti.unime.it (G.D.M.); mberretta@unime.it (M.B.); 4 Section of Legal Medicine, Department of Biomedical, Dental, Morphological and Functional Imaging Sciences, University of Messina, 98125 Messina, Italy; enzocianci.1997@gmail.com (V.C.); cristina.mondello@unime.it (C.M.)

**Keywords:** liquid biopsy, circulating tumor DNA, minimal residual disease, endometrial cancer, cervical cancer, HPV ctDNA, molecular profiling, recurrence surveillance, clinical validity, clinical utility

## Abstract

Endometrial and cervical cancers can recur from subclinical disease not evident on routine surveillance. This narrative review critically evaluates liquid biopsy for minimal residual disease (MRD) assessment and recurrence monitoring. Post-treatment human papillomavirus (HPV) circulating tumor DNA (ctDNA) in cervical cancer has the strongest disease-specific prospective evidence of clinical validity; persistent detection is strongly associated with recurrence, but moderate sensitivity and false-negative results do not support treatment de-escalation on negativity alone. With regard to endometrial cancer, perioperative ctDNA has prognostic support from an 11-study, 1298-patient meta-analysis and additional cohorts, although assay heterogeneity and limited independent replication constrain clinical readiness. Postoperative positivity generally shows stronger associations than preoperative detection. Cervicovaginal and urine DNA-methylation studies provide preliminary evidence for detecting established recurrence, especially local recurrence, but not prospective molecular lead time or clinical utility. Digital PCR, disease-specific fixed panels, tumor-informed assays, and error-corrected sequencing serve distinct settings; broad pan-cancer plasma profiling remains mainly an advanced-disease tool. Circulating tumor cells, extracellular vesicles, microRNAs, tumor-educated platelets, and fragmentomics remain exploratory. Liquid biopsy should remain an investigational adjunct until prospective trials show that acting on molecular findings improves outcomes.

## 1. Introduction

Endometrial cancer (EC) and cervical cancer (CC) are major gynecologic malignancies with distinct epidemiological patterns, etiologic drivers, and clinical care pathways. CC remains a major global health burden, particularly in low- and middle-income countries. By contrast, EC incidence and mortality are increasing in many settings, whereas the incidence of several other solid tumors has plateaued or declined [1].

Persistent infection with high-risk human papillomavirus (HPV) genotypes drives cervical carcinogenesis, and global outcome disparities largely reflect unequal access to HPV vaccination, screening, and treatment services; the World Health Organization estimates that approximately 94% of CC deaths in 2022 occurred in low- and middle-income countries [2]. In the United States, cancer statistics projected 13,490 new cases of invasive CC and 4200 deaths in 2026 [3]. The increasing burden of EC is associated with obesity-related unopposed estrogen exposure, population aging, and metabolic risk factors [4].

**Figure 1:**
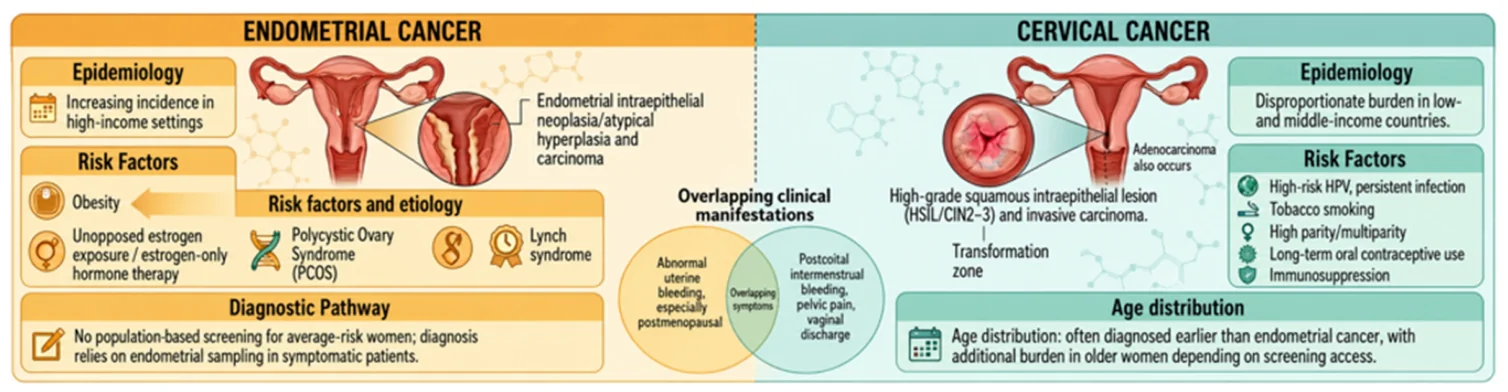
Comparative clinical and etiologic framework for endometrial and cervical cancer. The scheme contrasts major epidemiological patterns, risk factors, precursor lesions, and diagnostic pathways, while highlighting partially overlapping presenting symptoms. Endometrial cancer is primarily framed by obesity, unopposed estrogen exposure, polycystic ovary syndrome, and Lynch syndrome, with diagnosis relying on symptom-driven endometrial sampling in average-risk women. Cervical cancer is framed by persistent high-risk HPV infection, additional epidemiological and behavioral cofactors, and screening-dependent detection of precursor lesions at the transformation zone. An initial version of this illustration was generated with ChatGPT, version 5.6 (OpenAI, San Francisco, CA, USA); the authors edited, finalized, and scientifically verified the figure.

Figure 1 summarizes these epidemiological, etiologic, diagnostic, and clinical differences and their relevance to MRD-oriented liquid-biopsy strategies.

A clinically relevant proportion of patients experience recurrence despite curative-intent treatment. MRD denotes biologically persistent, subclinical malignant disease after treatment that is below the detection threshold of conventional assessment. A positive post-treatment liquid-biopsy result indicates molecular residual disease or molecular recurrence, whereas clinical recurrence requires confirmation by examination, imaging, pathology, or a combination thereof. Molecular detection may precede overt relapse, but it does not demonstrate that intervention improves outcomes [4,5].

Current diagnosis and surveillance rely on complementary modalities. Tissue biopsy and histopathology remain the reference standard for diagnosis, grading, histotype assignment, and tissue-based molecular classification. Cervical or vaginal cytology and HPV testing assess local epithelial or viral abnormalities in defined settings, whereas cross-sectional imaging provides anatomical staging, response assessment, and localization of suspected recurrence [6]. The 2026 Society of Gynecologic Oncology clinical practice statement identifies symptom review and physical examination as the most effective components of post-treatment surveillance across gynecologic cancers, discourages routine vaginal cytology in uterine-cancer surveillance, and regards ctDNA as an emerging strategy that requires additional study [6]. A single tissue sample, however, is spatially and temporally constrained and may not capture heterogeneity between the primary tumor and later metastatic clones [7].

Repeated surgical or percutaneous tissue sampling is invasive and is not suitable for frequent longitudinal monitoring. Cytology is comparatively simple and inexpensive but samples the local mucosal compartment rather than systemic microscopic disease; current CC guidelines do not recommend vaginal-vault cytology for routine recurrence detection, particularly after radiotherapy, and treatment-related atypia can complicate interpretation [8]. HPV testing is central to screening and triage and is used at defined intervals after fertility-sparing treatment, but it is not equivalent to measuring tumor-derived HPV DNA in plasma [2,8].

Serum CA125 may be considered selectively in EC follow-up when it was elevated at baseline or when clinically indicated, but it is not a validated stand-alone MRD assay. CT, MRI, and 18F-FDG PET/CT remain indispensable for anatomical assessment yet primarily detect macroscopic disease; small-volume deposits, inflammation, and post-treatment fibrosis can limit sensitivity or specificity [4,6,8,9].

Liquid biopsy provides molecular information that differs from the anatomical and histological information obtained by conventional assessment. By analyzing tumor-derived nucleic acids, cells, extracellular vesicles, or host-response signals in blood or regional biofluids, it can support serial molecular assessment and may reveal a recurrence-associated signal before radiographic confirmation in selected cohorts. It cannot localize disease and cannot replace histopathology, cytology/HPV testing, staging, or imaging [7,10,11].

Liquid biopsy is currently best regarded as an investigational adjunct for risk stratification and surveillance research. Table 1 compares conventional modalities with liquid-biopsy approaches and clarifies their distinct intended uses, strengths, and limitations.

## 2. Review Scope and Methodological Approach

This pathology-oriented narrative review examines liquid-biopsy approaches for MRD assessment and recurrence surveillance in EC and CC. Relevant sources were identified through PubMed/MEDLINE, Scopus, Web of Science, ClinicalTrials.gov, backward and forward citation chaining, and documents from the American Society of Clinical Oncology, U.S. Food and Drug Administration, Society of Gynecologic Oncology, ESGO/ESTRO/ESP, World Health Organization, and International Society for Extracellular Vesicles. PubMed/MEDLINE and ClinicalTrials.gov searches were current to 30 July 2026, and trial-registry information was checked on 1 August 2026. Search concepts combined endometrial, uterine, and cervical cancer or carcinoma terms with liquid-biopsy analytes and terms relating to MRD, prognosis, recurrence, survival, treatment response, and perioperative, preoperative, postoperative, postsurgical, or adjuvant settings. Both publication date and entry date [edat] fields were used in PubMed to improve the capture of recently indexed records. Study selection prioritized peer-reviewed English-language disease-specific evidence, particularly prospective or longitudinal cohorts, meta-analyses, clinical guidelines, and registered trials. Evidence was classified as post-treatment MRD or prognostic, or relating to contextual pretreatment or advanced disease, or protocol only. Preoperative detection studies, advanced-disease response-monitoring studies, and protocols without outcome data were not considered post-treatment MRD evidence. Explicitly documented shared populations were treated as overlapping, whereas similarities in institution, assay technology, sample size, or investigators were regarded as possible but unconfirmed overlap. Cross-tumor evidence was included only when relevant to analytical performance, regulatory context, or proof of concept and was identified as extrapolative. As a narrative review, this work did not involve prospective protocol registration, PRISMA reporting, duplicate independent screening, formal risk-of-bias assessment, or de novo quantitative synthesis; database-level retrieval and deduplication counts were not retained.

## 3. Biological Basis of Liquid-Biopsy Analytes

Liquid biopsy encompasses biologically distinct analytes rather than a single test. The principal classes considered here are ctDNA within the total cfDNA pool, circulating tumor cells (CTCs), extracellular vesicles (EVs), circulating RNAs and microRNAs, tumor-educated platelets (TEPs), DNA methylation signatures, and fragmentomic features. Each reflects different aspects of tumor shedding, cell viability, host response, or chromatin organization and therefore has a different level of analytical and clinical maturity [7,21].

### 3.1. Cell-Free DNA and Circulating Tumor DNA

ctDNA is the most extensively studied liquid-biopsy analyte for MRD across solid tumors. It constitutes a variable and often very small fraction of total plasma cfDNA. Apoptosis and necrosis are established sources of extracellular DNA, whereas active release remains a proposed mechanism. Neutrophil extracellular-trap formation (NETosis) can also increase the non-tumor cfDNA background [7,22]. Regulated cell-death pathways, including cuproptosis, are being investigated as potential sources of pathway-level biomarkers, although their relevance to gynecologic liquid-biopsy MRD remains undefined [23].

Tumor-derived fragments are often shorter than hematopoietic cfDNA. Fragmentomic studies have reported enrichment of ctDNA fragments around 134–144 base pairs compared with the approximately 166-base-pair mono-nucleosomal peak of much non-tumor cfDNA. Size selection and genome-wide fragmentation analysis can enrich tumor-derived signal, although performance is assay- and tumor-context dependent [24,25].

The central problem in early-stage gynecologic disease is molecular scarcity. Plasma HPV DNA was detected in 10.0% of early-stage and 63.3% of advanced CCs in one cohort, illustrating stage-dependent shedding [26]. At very low tumor fractions, analytical sensitivity is constrained not only by sequencing error but also by stochastic sampling and the finite number of genome equivalents in the collected plasma volume. Blood volume, cfDNA yield, sampling time, and assay design therefore affect detection probability, and input requirements reported for other tumor types cannot be generalized to gynecologic cancers [10,26]. Delayed processing can release leukocyte genomic DNA and dilute the signal, so prompt plasma separation or validated stabilizing tubes and standardized handling are required [27].

### 3.2. Circulating Tumor Cells

CTCs are intact malignant cells shed into the circulation. Unlike ctDNA, they can provide cellular morphology and material for single-cell genomic, transcriptomic, proteomic, or functional analyses. Their rarity, heterogeneous phenotype, and vulnerability to sampling error remain major limitations in EC and CC [28].

Epithelial-to-mesenchymal transition can reduce epithelial markers such as EpCAM, thereby limiting epithelial-marker-dependent capture. Marker-agnostic microfluidic, size-based, and deformability-based approaches can recover broader populations, but inter-platform concordance and disease-specific clinical thresholds remain insufficient for routine MRD use. CTC clusters may have distinct metastatic biology, yet most supporting evidence is cross-tumor and should not be extrapolated directly to gynecologic surveillance [29,30].

### 3.3. Extracellular Vesicles

EVs are lipid-bilayer-delimited particles released by cells and may carry DNA, RNA, microRNAs, lipids, and proteins. Consistent with the International Society for Extracellular Vesicles MISEV2023 recommendations, the umbrella term EV is preferable unless endosomal biogenesis has been demonstrated; exosome and small EV are not interchangeable terms [31,32]. Their protected cargo and potential reflection of cell state make EVs attractive biomarkers.

Evidence for EV-associated biomarkers in EC remains predominantly diagnostic. A 2025 systematic review included 23 studies. All of these studies evaluated diagnostic biomarkers, seven also assessed prognostic associations, but no prognostic biomarker was replicated across studies, and no study assessed EVs as monitoring, predictive, response, or safety biomarkers. Study quality, limited adherence to MISEV recommendations, and uncertainty as to whether candidate analytes were genuinely vesicle-associated rather than co-isolated were major limitations [33]. Translation is further limited by heterogeneous isolation methods, co-isolation of non-vesicular particles, and uncertain tumor-of-origin attribution [32,34].

### 3.4. Epigenetic Biomarkers and Tumor-Educated Platelets

DNA methylation and host-response analytes offer complementary information but remain less clinically mature than ctDNA mutation or HPV-sequence assays for post-treatment surveillance. Diagnostic studies have shown EC detection using urine, cervicovaginal self-samples, and cervical scrapes [35]. Cross-tumor experience with urinary methylation assays and cytology-integrated surveillance in bladder cancer illustrates the feasibility of non-invasive epigenetic monitoring, although these findings remain extrapolative and do not constitute direct evidence for gynecologic MRD [36,37]. Two disease-specific studies extend proximal-biofluid methylation into the post-treatment setting. In CC, 47 patients without recurrence provided paired pre- and post-treatment samples and 20 patients were sampled at confirmed recurrence; methylation positivity among recurrent cases was 77.8% in cervicovaginal self-samples and 65% in urine, with all 10 local recurrences positive in evaluable self-samples [38]. In EC, 43 patients without recurrence provided pre- and post-treatment samples and 17 patients were sampled at recurrence; methylation positivity was 62.5% in cervicovaginal self-samples and 58.8% in urine, reaching 100% and 90%, respectively, for local recurrences [39]. Both studies provide preliminary evidence for detection at established recurrence, but neither established prospective molecular lead time, assessed performance in an unselected asymptomatic surveillance cohort, or provided recommendations for treatment-guiding utility.

TEP RNA profiles have differentiated EC from benign gynecologic conditions in preliminary diagnostic studies, including machine-learning models achieving an area under the curve of 0.841, but the signal reflects an indirect host response and lacks validated recurrence thresholds [40,41].

Table 2 compares the principal liquid-biopsy analytes investigated in gynecologic oncology, highlighting their biological basis, potential clinical applications, and key limitations.

### 3.5. Clinical Readiness of Liquid-Biopsy Biomarkers

The clinical evidence differs substantially among biomarker classes. Post-treatment HPV ctDNA in CC has the strongest evidence for disease-specific prospective clinical validity, including multicenter chemoradiotherapy data, a 2026 longitudinal cohort, a 2026 meta-analysis, and complementary prospective cohorts [16,17,18,19,20]. Persistent positivity is highly specific and strongly prognostic, but moderate sensitivity and documented recurrence of false negatives mean that a negative result cannot support treatment de-escalation. Proximal-biofluid methylation has provided preliminary disease-specific evidence for detecting established cervical and endometrial recurrence, particularly local recurrence, but not for prospective lead time or treatment selection [38,39].

With regard to EC, an 11-study meta-analysis of 1298 patients and subsequent prospective and real-world studies support perioperative prognostic validity, although partial cohort overlap and methodological heterogeneity limit independent replication. Postoperative positivity generally shows stronger associations than preoperative detection, but assays, sampling windows, histologies, study designs, and patient overlap remain heterogeneous, and no ctDNA-directed intervention has established clinical utility [15,42,43,44,45,47]. Small studies using personalized junction assays, routine *TP53* monitoring, mixed gynecologic cohorts, and a disease-specific fixed panel provide feasibility data across different workflows [48,49,50,51]. A 93-record scoping review likewise characterized the evidence as promising but immature and insufficiently standardized [52]. Broad pan-cancer fixed-panel plasma profiling is used mainly to identify actionable alterations in advanced disease [53], whereas disease-specific fixed panels and tumor-informed personalized assays are distinct research strategies. CTCs, EVs, microRNAs, TEPs, and fragmentomics remain exploratory or diagnostic.

The 2026 ASCO guideline addresses somatic ctDNA but excludes viral DNA in virus-associated cancers, microRNAs, and extracellular-vesicle-associated nucleic acids; conclusions regarding HPV ctDNA and these analytes therefore require disease-specific evidence [53].

## 4. DNA-Based Analytical Platforms for MRD Detection

DNA-based MRD testing relies primarily on focused PCR methods or error-corrected sequencing, but methylation-aware sequencing and fragmentomics represent additional specialized approaches. Performance must be described at three distinct levels: analytical sensitivity or limit of detection, diagnostic sensitivity and specificity in a defined population, and clinical validity for association with outcome. Demonstrating any of these does not by itself establish clinical utility.

### 4.1. Polymerase Chain Reaction Methodologies

Digital PCR partitions a reaction into thousands of microcompartments and uses end-point fluorescence with Poisson modeling for absolute target quantification. ddPCR and related digital approaches are well suited to predefined somatic variants or viral sequences and generally provide higher analytical sensitivity than conventional qPCR for low-copy targets [54,55].

Focused digital assays can offer shorter analytical turnaround and lower per-sample laboratory resource requirements than customized broad sequencing, but they require prior target knowledge and have limited multiplexing. They may therefore miss newly emergent subclonal variants. Their total cost and turnaround remain laboratory- and workflow-dependent, and cost-effectiveness in gynecologic MRD has not been established [54].

### 4.2. Next-Generation Sequencing Platforms

NGS can interrogate multiple variants, copy-number changes, and, in specialized workflows, methylation or fragmentomic features. Error suppression can use unique molecular identifiers and integrated digital-error models; performance depends on DNA input, library architecture, sequencing depth, and bioinformatic implementation [56,57]. Broad tumor-uninformed pan-cancer panels can be deployed without customized tumor sequencing and are useful for plasma comprehensive genomic profiling in advanced or recurrent disease; they are not equivalent to deep-MRD assays [58]. Disease-specific fixed panels target recurrent alterations without patient-specific assay development and can support research recurrence detection, but their sensitivity remains constrained by panel coverage and low variant allele fractions [51]. Tumor-informed methods select patient-specific variants from tumor tissue, often with matched normal or leukocyte DNA depending on platform and workflow, improving signal-to-noise for longitudinal monitoring but requiring adequate tissue, assay-design time, and additional resources. Tracking a fixed personalized variant set can also miss newly arising resistance alterations.

### 4.3. Analytical and Clinical Performance of HPV ctDNA Assays

HPV-associated CC offers a tumor-linked viral target. HPV-seq uses broad viral capture and deep sequencing, whereas qPCR or dPCR uses predefined genotype-specific targets. Across the pooled pretreatment platform analysis, NGS-based methods showed higher detection sensitivity than ddPCR or qPCR, but this baseline diagnostic comparison should not be extrapolated to post-treatment MRD or prognostic performance [59]. Two 2026 studies provided additional platform-specific data. A small HPV-seq study enrolled 31 patients receiving concurrent chemoradiotherapy, with plasma evaluable in 27, and associated dynamic HPV-ctDNA decline with early treatment response [60]. In a separate 87-patient nanoplate dPCR study using tumor HPV status as the reference, plasma dPCR detected 82 of 83 HPV16/18/31-positive cancers and was negative in all four tumor-HPV-negative cancers; all 20 healthy-donor controls were negative in a separate experiment [61]. Primary head-to-head work further showed that HPV-seq detected ctDNA at lower copy levels and retained broader genotype information than genotype-targeted dPCR [62]. Interpretation is limited by genotype restriction, the four-patient tumor-negative denominator, heterogeneous sampling, and few recurrence events; the studies do not provide population-level diagnostic specificity or comparative evidence for post-treatment MRD monitoring or treatment guidance.

Across a meta-analysis reporting 36 pretreatment studies and 2986 patients, platform-specific analyses comprised 7 NGS, 19 ddPCR, and 11 qPCR study-level datasets; these counts were non-mutually exclusive because Leung et al. [62] contributed both dPCR and NGS data. Pooled sensitivity was 0.94 for NGS, 0.81 for ddPCR, and 0.51 for qPCR; pooled specificity was 0.95, 0.98, and 0.93, respectively [59]. These estimates derive from heterogeneous baseline-detection studies across HPV-associated cancers and should not be interpreted as post-treatment MRD performance. In the prospective CC validation cohort, HPV-seq and dPCR produced similar prognostic performance despite different analytical characteristics [16]. Table 3 summarizes the intended setting, analytical characteristics, and supporting evidence for each platform.

**Figure 2:**
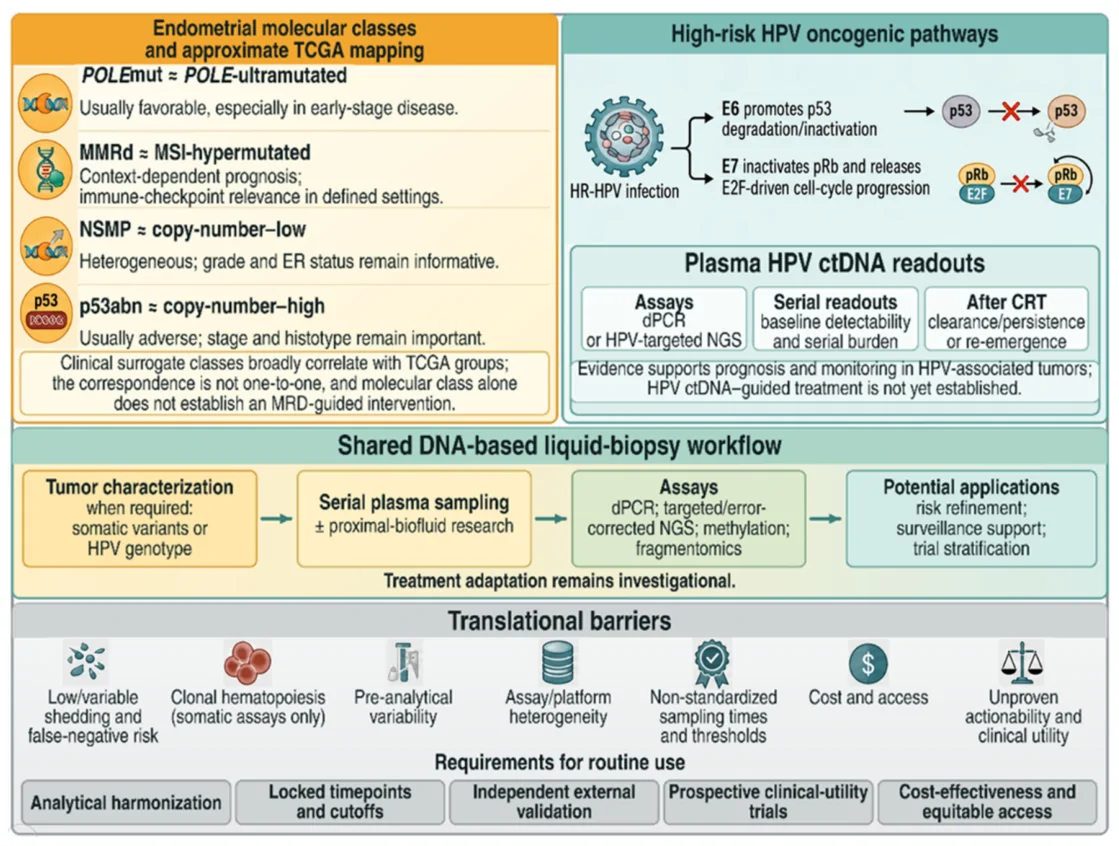
Endometrial molecular classification, high-risk HPV oncogenic signaling, and translational framework for DNA-based liquid biopsy in endometrial and cervical cancers. The upper-left panel illustrates the broad, non-identical correspondence between the clinically applied molecular classes of endometrial carcinoma and The Cancer Genome Atlas groups: pathogenic *POLE* exonuclease-domain mutant (*POLE*mut) tumors broadly correspond to *POLE*-ultramutated cancers, mismatch repair-deficient tumors to microsatellite instability-high cancers, no specific molecular profile tumors to copy-number-low cancers, and p53-abnormal tumors to copy-number-high cancers. Prognosis remains influenced by disease stage, histotype, grade, and estrogen-receptor status; molecular classification alone does not define a minimal-residual-disease-guided intervention. The upper-right panel summarizes the principal oncogenic pathways driven by persistent high-risk human papillomavirus infection. The E6 oncoprotein, through E6-associated protein, promotes p53 ubiquitination and degradation, whereas E7 promotes retinoblastoma-protein degradation or inactivation and releases E2F transcription factors, facilitating cell-cycle progression. Plasma human papillomavirus circulating tumor DNA can be assessed using digital polymerase chain reaction or human papillomavirus-targeted next-generation sequencing. Baseline detectability, serial molecular burden, and clearance, persistence, or re-emergence after chemoradiotherapy may provide prognostic and monitoring information, although treatment guided by human papillomavirus circulating tumor DNA has not been established. The central panel outlines a shared DNA-based liquid-biopsy workflow comprising tumor characterization when required, serial plasma sampling with optional proximal-biofluid research, molecular analysis, and potential applications in risk refinement, surveillance support, and clinical-trial stratification. The lower panel summarizes the principal translational barriers, including low or variable tumor-DNA shedding and false-negative risk, clonal hematopoiesis in somatic assays, pre-analytical variability, assay and platform heterogeneity, non-standardized sampling times and thresholds, cost and access, and unproven actionability and clinical utility. Requirements for routine implementation include analytical harmonization, predefined sampling time points and positivity cutoffs, independent external validation, prospective clinical-utility trials, and demonstration of cost-effectiveness and equitable access. Treatment adaptation based solely on liquid-biopsy findings remains investigational. Abbreviations: CRT, chemoradiotherapy; ctDNA, circulating tumor DNA; DNA, deoxyribonucleic acid; dPCR, digital polymerase chain reaction; E2F, E2F family of transcription factors; E6 and E7, high-risk human papillomavirus early oncoproteins 6 and 7; E6AP, E6-associated protein; ER, estrogen receptor; HPV, human papillomavirus; HR-HPV, high-risk human papillomavirus; MMRd, mismatch repair-deficient; MRD, minimal residual disease; MSI-H, microsatellite instability-high; NGS, next-generation sequencing; NSMP, no specific molecular profile; *POLE*, DNA polymerase epsilon catalytic subunit; *POLE*mut, pathogenic *POLE* exonuclease-domain mutant; p53abn, p53-abnormal; pRb, retinoblastoma protein; TCGA, The Cancer Genome Atlas. An initial version of this illustration was generated with ChatGPT, version 5.6 (OpenAI, San Francisco, CA, USA); the authors edited, finalized, and scientifically verified the figure.

Figure 2 summarizes the approximate correspondence between endometrial molecular classes and TCGA groups, the principal high-risk HPV oncogenic pathways in CC, and the potential applications and current implementation barriers of a shared DNA-based liquid-biopsy workflow.

## 5. Clinical Evidence and Applications in EC

EC is molecularly and histologically heterogeneous [4,13,63]. More broadly, endometrial pathology encompasses a spectrum of non-neoplastic and neoplastic conditions, including endometriosis-associated neoplasia [64,65]. Contemporary risk assessment integrates histotype, grade, stage, lymphovascular space invasion, and molecular features, including *POLE* mutation, MMR deficiency, p53 abnormality, and NSMP status [4,13,63]. Tissue-based classification remains essential for interpreting predictive biomarkers, including those relevant to immunotherapy [63,66]. Liquid biopsy may provide longitudinal information on selected tumor-derived signals but cannot reproduce full histomorphologic assessment or comprehensive tissue profiling [67].

### 5.1. Preoperative Risk Stratification and Prognostication

Preoperative cfDNA concentration and plasma ctDNA detectability have been associated with higher grade, deeper myometrial invasion, lymphovascular space invasion, advanced stage, and adverse outcomes in exploratory EC cohorts [68,69]. In an 83-patient international multicenter study, an off-the-shelf pan-cancer panel detected ctDNA in 16 patients (19.3%); recurrence occurred in 37.5% of ctDNA-positive versus 11.9% of ctDNA-negative patients, and positivity remained independently associated with recurrence (HR, 5.49; 95% CI, 1.5–20). However, 8 of the 14 patients with recurrent disease were ctDNA-negative at diagnosis, demonstrating that preoperative negativity does not rule out future relapse [43].

A 2026 meta-analysis of 11 studies and 1298 patients associated preoperative ctDNA positivity with worse progression-free survival (HR, 3.69; 95% CI, 2.58–5.26) and postoperative positivity with an even stronger association (HR, 12.61; 95% CI, 8.78–18.13) [42]. Some individual cohorts may also have been represented in the pooled estimate and were not interpreted as independent validation. In the prospective CODEC study, preoperative ctDNA correlated with PET- and MRI-defined tumor volume but not with relapse-free survival, indicating that the prognostic value of baseline detection depends on the clinical setting and assay [45]. Consistent with the 2026 ASCO guidelines, fractional or concentration-based ctDNA/cfDNA measures should not be used as generic surrogate measures of disease burden without a validated, setting-specific interpretation [53].

### 5.2. Postoperative MRD Monitoring and Recurrence Dynamics

Surgery remains central for most localized ECs, but postoperative clinicopathologic risk groups do not perfectly distinguish cure from occult residual disease. In a 2024 real-world study of stage I uterine malignancies, 101 patients contributed 267 plasma samples and median follow-up was 6.8 months. Tumor-informed ctDNA positivity was associated with shorter recurrence-free survival at the first postoperative time point (HR, 6.2; *p* = 0.0006) and during longitudinal monitoring (HR, 15.5; *p* < 0.0001). Recurrence occurred in 58% and 52% of ctDNA-positive patients at the corresponding analyses versus 6% and 0% of ctDNA-negative patients. The cohort was assay-specific and histologically heterogeneous, with high-risk carcinomas and sarcomas represented; it supports prognostic validity but not routine treatment selection [15].

A subsequent retrospective, multicenter real-world analysis included 61 patients with stage I–II uterine cancer and 233 plasma time points. ctDNA positivity was associated with reduced recurrence-free survival postoperatively (HR, 7.6; *p* = 0.003) and after definitive therapy (HR, 25.4; *p* = 0.0009); all patients with available outcome data who recurred were ctDNA-positive before or at recurrence, whereas none of the serially negative patients relapsed during reported follow-up [47]. This report and the earlier 101-patient study used the same commercial tumor-informed platform and had substantial investigator overlap; the extent of cohort independence is not fully clear. Both Signatera-based reports included substantial assay-provider participation and disclosed employment and equity relationships, limiting their external generalizability and necessitating independent validation [15,47].

Prospective perioperative studies support the importance of sampling time. In a 36-patient high-risk cohort, ctDNA obtained approximately 10 weeks after surgery was associated with recurrence (HR, 3.32; 95% CI, 1.05–10.51) and death (HR, 5.97; 95% CI, 1.11–36.08), whereas preoperative ctDNA was not significantly prognostic [44]. CODEC enrolled 50 patients with type 2 EC; 30 underwent ctDNA analysis and 25 had evaluable samples at 2–6 weeks. Detection at 24 h and 2–6 weeks was associated with poorer relapse-free survival, whereas preoperative and intraoperative detection were not, making the 2–6-week window hypothesis-generating but not yet validated for treatment selection [45]. Across studies, postoperative positivity appears more prognostic than preoperative detection, but no randomized ctDNA-directed intervention has shown clinical utility.

### 5.3. Serial Monitoring and Detection of Recurrence

Earlier personalized studies reported that serial ctDNA can track treatment response and precede imaging in selected EC cases [70]. A junction-based qPCR program developed assays from 36 high-risk cases; among 17 patients monitored serially, ctDNA detection preceded clinical recurrence in 5 of 8 recurrent cases and was concurrent in 1, although imaging was rarely synchronized with blood collection [48]. Because this report shares the institution, junction technology, sample size, and several investigators with the De Vitis study, the two reports cannot be assumed to represent fully independent cohorts. In a separate mixed cohort of 44 patients (24 endometrial, 17 ovarian, 2 synchronous endometrial/ovarian, and 1 endocervical adenocarcinoma), only four recurrent cases were followed through relapse; ctDNA preceded clinical, radiologic, or biomarker progression by 2–5 months in three, so the finding is supportive but not endometrial-specific [50].

In 21 patients with high-grade *TP53*-mutated EC, baseline ctDNA positivity was associated with 2-year recurrence-free survival of 18% versus 60% and overall survival of 40% versus 78%; postoperative persistence or reappearance commonly accompanied progression, with ctDNA preceding imaging in four cases and coinciding in one [49]. An EC-specific fixed panel detected hotspot variants in 10 of 14 patients sampled with established recurrence and in 1 of 25 without recurrence (sensitivity, 71.4%; specificity, 96%; accuracy, 87.2%); because sampling was cross-sectional at known recurrence, the study did not establish asymptomatic lead time [51]. A 93-record systematic scoping review concluded that cfDNA/ctDNA evidence across diagnosis, profiling, prognosis, and surveillance remains promising but immature, heterogeneous, and insufficiently standardized [52].

### 5.4. Alternative Biofluids: Diagnostic and Preliminary Recurrence-Detection Evidence

Peripheral-blood sensitivity may be limited in localized EC, and clonal hematopoiesis can generate plasma variants unrelated to the tumor [71]. Uterine lavage directly samples the endometrial cavity and can enrich tumor-associated alterations, but driver mutations may also occur in women without histopathologic malignancy [72]. Cervicovaginal self-samples and cervical scrapes combined with methylation markers have shown promising primary-detection performance [35]. In the 2026 post-treatment study, methylation was positive at established recurrence in 10 of 16 evaluable cervicovaginal self-samples and 10 of 17 urine samples, with the highest detection for local recurrence [39]. Sampling at established recurrence and reuse of models developed for primary detection preclude estimates of asymptomatic lead time or performance in an unselected longitudinal cohort and do not define a management algorithm.

## 6. Clinical Evidence and Applications in CC

Most CCs are driven by persistent high-risk HPV infection. The viral genome is absent from the human germline genome, making tumor-associated HPV sequences a biologically specific target; however, HPV detection in a mucosal sample can reflect infection without invasive cancer. Plasma HPV ctDNA therefore requires an assay, specimen, and clinical context that distinguish tumor-derived circulating signal from local infection [26,73]. Compared with tracking heterogeneous patient-specific somatic variants, a known tumor-associated HPV genotype can simplify focused longitudinal monitoring. Plasma HPV ctDNA should be interpreted alongside conventional staging and nodal assessment, including histopathologic or molecular evaluation of sentinel lymph nodes in selected settings [74].

### 6.1. Prognostic Significance After Definitive Chemoradiotherapy

A 2026 systematic review and meta-analysis identified 20 eligible studies, including 11 diagnostic and 6 prognostic studies. Pretreatment HPV cfDNA detection had pooled sensitivity of 0.47 and specificity of 0.96; these diagnostic estimates should not be extrapolated to post-treatment MRD. HPV cfDNA positivity at 3 months after treatment was associated with shorter progression-free survival (HR, 8.50; 95% CI, 4.69–15.41; I^2^ = 0%) [18]. Potential overlap between the meta-analysis and individual cohorts was considered when interpreting the evidence.

In the prospective BioRAIDs cohort, baseline serum HPV ctDNA was detected in 59 of 94 (63%) HPV16/18-positive CCs but was not associated with progression-free survival. End-of-treatment samples were available for 40 patients; 11 were positive, and end-of-treatment positivity was associated with shorter progression-free survival on multivariable analysis (HR, 14.25; 95% CI, 3.10–61.57). Among the eight patients with longitudinal follow-up after a positive end-of-treatment result, six remained positive while clinically disease-free and all six subsequently relapsed; in these persistently positive patients, the median interval from HPV ctDNA detection to clinical relapse was 10 months (range, 2–15 months) [19]. In a separate prospective cohort of 66 patients receiving definitive chemoradiation, including 17 who also received PDS0101, clearance during weeks 1, 3, and 5 was not associated with recurrence-free survival, whereas status at 3–4 months was prognostic: 2-year recurrence-free survival was 92.9% after clearance versus 30.0% with persistent detection (univariable HR, 7.21; 95% CI, 1.79–28.96), with a median follow-up of 23 months [20]. Genotype restriction, incomplete post-treatment sampling, mixed treatment exposure, and small positive follow-up subsets limit generalizability.

Curative treatment for locally advanced CC is based on external-beam radiotherapy with concurrent platinum-based chemotherapy followed by image-guided brachytherapy, using intracavitary and/or interstitial techniques as anatomically appropriate [8,75]. In the United States, pembrolizumab with chemoradiotherapy is FDA-approved for FIGO 2014 stage III–IVA disease; this jurisdiction-specific treatment development does not establish ctDNA-guided management [76]. In the prospective multicenter validation study NCT03853915, dPCR-detectable HPV ctDNA was associated with 2-year progression-free survival of 51% versus 77% at the end of chemoradiotherapy, 15% versus 82% at 4–6 weeks, and 24% versus 82% at 3 months for positive versus negative results, respectively. Molecular detection preceded clinical or radiographic recurrence by a median of 5.9 months [16]. The 5.9-month median lead time supports prognostic validity, but whether treatment initiated solely on ctDNA positivity improves survival remains unknown. Positive results therefore require clinical confirmation and, where appropriate, evaluation within biomarker-driven trials.

In a 141-patient NGS cohort spanning primary surgery, surgery plus adjuvant treatment, and primary oncologic treatment, positivity at the first post-treatment sample was associated with recurrence (adjusted HR, 19.1) and yielded sensitivity of 45%, specificity of 98%, positive predictive value of 83%, and negative predictive value of 90%. Considering any post-treatment positivity increased sensitivity to 61%, with specificity of 96%, positive predictive value of 78%, and negative predictive value of 92%. Among 23 patients who recurred and had post-treatment samples, circulating HPV DNA preceded clinical evidence in 14 (60.9%), with a mean lead time of 144 days, but remained negative throughout post-treatment follow-up in nine. These findings support a high-specificity rule-in and prognostic role, but the authors explicitly concluded that negativity is insufficient for treatment de-escalation [17]. The smaller HPV-seq and nanoplate dPCR studies reported dynamic treatment-response associations and exploratory longitudinal observations, but they had limited sample sizes, heterogeneous sampling schedules, or few recurrence events [60,61]. None of these studies evaluated whether biomarker-triggered treatment improves clinical outcomes.

A separate 2025 study evaluated home-collected cervicovaginal and urine samples in 47 CC patients without recurrence and 20 patients sampled at confirmed recurrence. DNA-methylation positivity among recurrent cases was 77.8% in evaluable cervicovaginal self-samples and 65% in urine; all 10 local recurrences were methylation-positive in self-samples. These data concern detection at established recurrence and do not establish prospective lead time or clinical utility [38].

### 6.2. Correlative ctDNA Analyses in Immunotherapy Trials and Ongoing Studies

HPV oncogene expression provides a rationale for immune-checkpoint blockade, but liquid biopsy is not an established substitute for tissue or validated clinical biomarkers of immunotherapy response [77]. The parent phase III CALLA trial randomized 770 patients to durvalumab or placebo with and after chemoradiotherapy. Durvalumab did not significantly improve progression-free survival in the biomarker-unselected population (HR, 0.84; 95% confidence interval, 0.65–1.08; *p* = 0.17) [75]. Because the parent trial did not improve progression-free survival, the subsequent ctDNA findings remain correlative.

In the CALLA ctDNA analysis, higher baseline burden and persistent post-treatment tumor-informed ctDNA were associated with progression or death, whereas clearance correlated with better outcomes [78]. These are prognostic correlative findings; treatment was not assigned according to ctDNA status, and the analysis does not demonstrate that ctDNA-guided immunotherapy improves outcomes.

Four registered studies address observational monitoring, feasibility of treatment de-escalation, shared decision-making, or biomarker integration. NCT05531981 is an observational serial HPV *E7* ctDNA registry record with estimated enrollment of 350 participants; it was last verified in August 2022 and last updated in October 2022, so its current recruitment status cannot be confirmed from the registry record [79]. NCT06878196 is a not-yet-recruiting, single-group study of approximately 20 patients with stage IVB or recurrent CC, designed primarily to explore the feasibility and accuracy of ctDNA-guided immunochemotherapy de-escalation [80]. NCT07270666 is a recruiting, non-randomized pragmatic pilot of 30 patients with advanced or recurrent MMRd/MSI-H EC; its primary endpoint is the feasibility of ctDNA-informed shared decision-making about discontinuing maintenance immune-checkpoint inhibition after one year of a standard first-line regimen combining chemotherapy with an immune-checkpoint inhibitor, not comparative efficacy [81]. NCT07061977 is a recruiting randomized phase III CC trial in which baseline and 3-month ctDNA are integrated prognostic or predictive biomarkers, but ctDNA does not determine treatment allocation [82]. None have yet reported evidence that ctDNA-guided management improves clinical outcomes.

## 7. Complementarity of Liquid Biopsy, Imaging, and Radiomics

Liquid biopsy provides a systemic molecular signal but no anatomical localization. MRI, CT, and 18F-FDG PET/CT localize macroscopic disease and remain essential for staging, treatment planning, and evaluation of suspected recurrence, yet very small deposits and post-treatment inflammation or fibrosis can be difficult to characterize [14,83]. A positive ctDNA result therefore cannot identify which lymph node or organ is involved, and a negative result cannot exclude disease when shedding or assay sensitivity is limited.

Radiomics can extract quantitative spatial and textural features from standard imaging. Combining radiomic features with ctDNA, methylation, or fragmentomic data could integrate anatomical and molecular information [84,85]. Such models remain exploratory and require prospective external validation of prespecified algorithms. An older mixed gynecologic-cancer cohort reported molecular detection before CT in a small subset, but its histologic heterogeneity and sample size preclude disease-specific treatment recommendations [86].

## 8. From Prognostic Association to Clinical Utility

Clinical validity refers to association with recurrence or outcome, whereas clinical utility requires evidence that biomarker-guided management improves patient-centered outcomes; the latter has not been established for MRD-directed treatment in EC or CC. Molecular lead time can be beneficial only if it leads to an effective, safe, and appropriately timed intervention rather than anxiety, unnecessary imaging, biopsy, or overtreatment.

### 8.1. Potential Escalation Strategies

Persistent postoperative or post-treatment ctDNA has been associated with increased relapse risk across endometrial/uterine and cervical cohorts [15,16,17,44,45,47]. Current investigational applications include prognostic counseling, clinically indicated confirmatory assessment or closer surveillance, and enrollment in interventional trials. A positive result should not automatically trigger systemic therapy when imaging is negative, because the optimal threshold, repeat-testing rule, intervention, and balance of benefit and harm are unknown.

### 8.2. Treatment De-Escalation and Avoidance of Unnecessary Toxicity

Serial ctDNA negativity or clearance has been associated with favorable outcomes in several cohorts, but there are no prospective randomized data showing that adjuvant chemotherapy, radiotherapy, or maintenance immunotherapy can be safely omitted solely because MRD is negative in EC or CC. The 2026 cervical cohort directly demonstrated that post-treatment negativity had insufficient sensitivity and negative predictive value for safe de-escalation [17]. The randomized DYNAMIC trial in stage II colon cancer provides cross-tumor proof of concept that ctDNA-guided management can reduce chemotherapy use without compromising recurrence-free survival, but it cannot be directly extrapolated to gynecologic disease [87]. De-escalation must therefore be tested in disease-specific prospective trials with predefined non-inferiority margins and safeguards against false-negative results.

## 9. Barriers to Clinical Implementation

Routine adoption will require reliable performance at low tumor burden, standardized assays, evidence of clinical benefit, feasibility, and equitable access.

### 9.1. Biological and Analytical Limitations

Low shedding in localized disease creates a fundamental false-negative risk even for error-corrected assays. Sampling time, plasma volume, cfDNA input, tumor vascularity, treatment-related changes, and platform design can influence detection. Clinically relevant false negatives were observed in both preoperative endometrial and post-treatment cervical cohorts [17,43]. Clonal hematopoiesis can produce non-tumor variants at low variant allele fractions; parallel analysis of matched leukocyte DNA is valuable in many low-VAF workflows, although requirements vary by assay and intended use [57,71,88]. Pre-analytical deviations, failed tissue retrieval, insufficient tumor material, and non-informative personalized assay design can delay or prevent reporting. False-positive or discordant results can cause anxiety and cascades of imaging or biopsy, whereas false-negative results can provide false reassurance.

### 9.2. Standardization, Regulation, Access, and Reimbursement

Implementation requires validated collection tubes and processing intervals, extraction controls, analytical limits of detection and limits of blank, quality-assurance materials, bioinformatic version control, transparent reporting of non-informative results, and external proficiency testing [57,89]. Within its scope of somatic ctDNA, the 2026 ASCO guideline recommends testing outside trials when a result supports a specific evidence-based action, when tissue testing is not feasible or timely, or when an approved indication permits or requires plasma testing. The ASCO recommendation to consider tissue confirmation after negative, inconclusive, or clinically discordant plasma genotyping applies to somatic tumor genotyping and not to all MRD assays [53]. In May 2026, the U.S. FDA approved atezolizumab after cystectomy for muscle-invasive bladder cancer with ctDNA-defined MRD and authorized Signatera CDx as the companion diagnostic for that indication [90]. This indication- and assay-specific authorization does not extend to EC or CC. Practical adoption will also depend on local laboratory capacity, tissue logistics, turnaround time, repeat-test schedules, reimbursement, out-of-pocket cost, and setting-specific cost-effectiveness; robust gynecologic health-economic evidence is currently insufficient.

## 10. Conclusions and Future Directions

Evidence for liquid biopsy in gynecologic cancer remains heterogeneous and insufficient for routine stand-alone use. Post-treatment HPV ctDNA after definitive CC therapy has the strongest disease-specific prospective clinical-validity evidence, supported by prospective cohorts and a 2026 meta-analysis; persistent detection is associated with recurrence risk and molecular lead time [16,17,18,19,20]. In one 141-patient longitudinal cohort, sensitivity for recurrence was 45% at the first post-treatment sample and 61% when any post-treatment positivity was considered; 9 of 23 patients who recurred and had post-treatment samples remained negative throughout follow-up. Accordingly, negativity alone should not be used to support treatment de-escalation [17].

Regarding EC, an 11-study, 1298-patient meta-analysis together with prospective perioperative, longitudinal, and recurrence-detection studies supports prognostic validity, but assay heterogeneity and limited independent replication remain important limitations. Postoperative positivity generally shows stronger prognostic associations than preoperative detection, but assay architecture, sampling windows, histologic composition, cohort overlap, and outcome definitions remain variable; no ctDNA-directed trial has established treatment-guiding utility [15,42,43,44,45,47,48,49,51,52].

Exploration remains ongoing with regard to CTCs, microRNAs, TEPs, and fragmentomics for MRD surveillance. Endometrial EV evidence remains predominantly diagnostic, and no validated EV monitoring biomarker has been established [33]. Proximal-biofluid DNA methylation has preliminary disease-specific evidence for detecting established cervical and endometrial recurrence, especially local recurrence, but not for prospective asymptomatic lead time or treatment guidance [38,39]. Analytical performance, detection at established recurrence, and association with outcome should not be conflated with evidence that biomarker-guided treatment improves survival. No randomized evidence currently supports routine escalation or de-escalation of EC or CC treatment solely on the basis of a positive or negative liquid-biopsy result.

Prospective disease-specific trials should prespecify sampling windows, assays, quality-control procedures, handling of failed, discordant, and false-negative results, and management algorithms linked to clinically meaningful endpoints. They should also assess access, turnaround time, reimbursement, cost-effectiveness, patient-reported outcomes, and downstream harms. Until such evidence is available, liquid biopsy should be interpreted within multidisciplinary workflows and used primarily in research, validated genomic-profiling indications, or carefully defined clinical scenarios rather than as a stand-alone surveillance test.

## Figures and Tables

**Table 1:** Comparison of conventional diagnostic and surveillance modalities with liquid-biopsy approaches in endometrial and cervical cancer.

Modality	Principal Role	Key Strengths	Limitations for MRD/Recurrence Surveillance
Tissue biopsy and histopathology [6,7,12,13]	Diagnosis; grading and histotype; tissue-based molecular classification.	Reference standard; preserves architecture and spatial context.	Invasive and site-specific; unsuitable for frequent serial sampling; may miss spatial or temporal heterogeneity.
Cervical/vaginal cytology [6,8]	Screening and local epithelial assessment; limited guideline-defined follow-up.	Simple, inexpensive, broadly available, and directly evaluates cell morphology.	Local mucosal sample only; not recommended for routine invasive-cancer recurrence detection after definitive treatment; post-radiotherapy atypia may confound interpretation.
HPV testing [2,8]	Screening, triage, and defined post-treatment pathways for precancer or fertility-sparing management.	Sensitive, scalable detection of oncogenic HPV.	Local positivity may reflect infection; it is not equivalent to plasma tumor-derived HPV ctDNA and is not a systemic MRD assay.
Serum biomarker (CA125) [4,6]	Adjunctive assessment in selected EC contexts, particularly when elevated at baseline or clinically indicated.	Rapid, familiar, and broadly accessible.	Limited sensitivity and specificity for microscopic recurrence; benign conditions may elevate values; no stand-alone MRD role.
CT, MRI, and 18F-FDG PET/CT [4,8,14]	Staging, anatomical localization, response assessment, and suspected recurrence.	Essential spatial information and confirmation of macroscopic disease.	Limited for very small-volume deposits; inflammation and fibrosis may confound interpretation; no molecular readout.
Liquid biopsy (ctDNA/HPV ctDNA; dPCR/NGS) [10,15,16,17,18,19,20]	Serial molecular risk assessment and investigational MRD monitoring.	Minimally invasive and repeatable; may provide systemic molecular lead time.	Low shedding, clonal hematopoiesis, assay heterogeneity, failed/non-informative tests, access and cost barriers, and no validated threshold for routine clinical action.

**Table 2:** Comparative readiness of liquid-biopsy analytes in gynecologic oncology.

Liquid-Biopsy Analyte	Biological Source or Molecular Signal	Potential Application	Principal Limitations
Somatic ctDNA (mutation-based) [15,42,43,44,45]	Tumor-derived fraction of plasma cfDNA; abundance depends on shedding, treatment timing, clearance, and assay design.	Perioperative and postoperative EC risk stratification; patient-specific longitudinal monitoring.	Very low abundance in localized disease; preoperative false negatives; clonal hematopoiesis; heterogeneous assays and sampling windows; no treatment-guiding threshold.
HPV ctDNA/circulating HPV DNA [16,17,18,19,20]	Tumor-associated viral sequences released into plasma from HPV-driven cancer.	Post-treatment CC risk stratification and investigational molecular recurrence monitoring.	Applicable only to HPV-associated disease; moderate surveillance sensitivity and documented false negatives; no validated threshold for treatment de-escalation or escalation.
Circulating tumor cells (CTCs) [28,29,46]	Intact malignant cells entering blood from primary or metastatic sites.	Single-cell molecular analysis and functional research.	Very rare; phenotype-dependent capture; epithelial-marker loss during EMT; limited disease-specific standardization.
Extracellular vesicles (EVs) [32,33,34]	Lipid-bilayer-delimited particles carrying nucleic acids, proteins, and lipids; subtype biogenesis is often unproven.	Predominantly diagnostic biomarker research; limited, unreplicated prognostic associations.	Heterogeneous isolation and characterization; co-isolates; uncertain tumor origin; limited MISEV adherence; no validated monitoring or MRD role.
Tumor-educated platelets (TEPs) [40,41]	Host platelets with tumor-associated RNA-content or splicing changes.	Preliminary diagnostic classification and host-response profiling.	Indirect signal; systemic confounding; limited specificity; no validated recurrence utility.
DNA methylation [35,38,39]	Epigenetic alterations measured in plasma or proximal biofluids, including cervicovaginal self-samples and urine.	Primary-detection research and preliminary disease-specific detection at established cervical and endometrial recurrence, especially local recurrence.	Marker/model and specimen dependence; current recurrence evidence is small and not longitudinally validated for lead time or treatment guidance.
EV-associated RNA signatures [33]	RNA patterns measured in EV preparations derived from plasma, serum, or other biofluids; vesicular encapsulation was not established consistently.	Exploratory diagnostic and prognostic biomarker research; no validated monitoring, predictive, response, or safety application.	Pre-analytical and normalization variability; uncertain tumor origin; mainly retrospective, bioinformatic, or small-cohort evidence.
Fragmentomics [24]	cfDNA fragment-length distributions and genome-wide fragmentation patterns.	Tumor-signal enrichment.	Bioinformatic complexity, platform dependence, uncertain performance at very low tumor fractions, and limited prospective gynecologic validation.

**Table 3:** Comparative analytical characteristics and evidence context of DNA-based liquid-biopsy platforms relevant to endometrial and cervical cancer. Reported performance is platform-, specimen-, and study-specific and does not alone establish treatment-guiding utility.

Platform	Analytical Design			Principal Setting	Strengths		Limitations/Evidence Caveats
qPCR [59]	Targeted amplification with calibration curves.	Known HPV genotype or higher-copy predefined target.		Rapid, widely available, and operationally simple.			Lower pooled pretreatment sensitivity than dPCR/NGS in heterogeneous HPV-associated cancer studies; target-, matrix-, and inhibitor-dependent.
ddPCR/dPCR [16,54,61]				Target partitioning with end-point absolute quantification.	Predefined somatic variants or HPV sequences; serial focused monitoring.	High analytical sensitivity and short analytical turnaround; tumor–plasma HPV concordance was 82/83 among HPV16/18/31-positive cancers and 4/4 among tumor-HPV-negative cancers; 20/20 healthy donors were negative in a separate control experiment.	Limited multiplexing and discovery; prior target knowledge required; genotype restriction, the four-patient tumor-negative denominator, heterogeneous longitudinal sampling, and only four relapses limit generalizability; no proven post-treatment MRD or prognostic superiority to HPV-seq.
	Broad pan-cancer fixed-panel plasma CGP [53,58]	Off-the-shelf panel of recurrent cancer-associated alterations.	Plasma comprehensive genomic profiling, mainly in advanced/recurrent disease.	Broad actionable-alteration coverage without patient-specific assay development.			Not optimized for ultra-low-burden MRD; clonal hematopoiesis and background variants; negative or discordant plasma genotyping may require tissue confirmation when clinically relevant.
Disease-specific fixed-panel NGS [51]	Fixed panel of recurrent EC hotspot alterations without patient-specific assay design.			Research detection of established EC recurrence.	Standardized disease-focused workflow; one small study reported 71.4% sensitivity, 96% specificity, and 87.2% accuracy.		Panel coverage and limit of detection are study-specific; 4 of 14 recurrences were missed; cross-sectional testing at known recurrence does not establish lead time or clinical utility.
Tumor-informed error-corrected NGS [15,47,56,58]	Patient-specific variants selected from tumor tissue, often with matched normal or leukocyte DNA depending on platform and workflow.	Postoperative and longitudinal MRD research.		Improved patient-specific signal-to-noise for longitudinal monitoring.			Requires tumor tissue, custom development, longer initial turnaround, and more resources; assay design can be non-informative.
HPV-seq viral capture [16,17,62]	Broad viral-genome capture and deep sequencing.			HPV-associated genotyping and MRD research.	Broad genotype coverage, high analytical sensitivity, and prospective/longitudinal monitoring data.		HPV-associated tumors only; platform-specific workflows; no proven treatment-guiding superiority over dPCR.

## Data Availability

No new data were created or analyzed in this study. Data sharing is not applicable to this article.
